# Polybrene induces neural degeneration by bidirectional Ca^2+^ influx-dependent mitochondrial and ER–mitochondrial dynamics

**DOI:** 10.1038/s41419-018-1009-8

**Published:** 2018-09-20

**Authors:** Feixiang Bao, Hongyan Shi, Mi Gao, Liang Yang, Lingyan Zhou, Qiuge Zhao, Yi Wu, Keshi Chen, Ge Xiang, Qi Long, Jingyi Guo, Jian Zhang, Xingguo Liu

**Affiliations:** 1 CAS Key Laboratory of Regenerative Biology, Joint School of Life Sciences, Guangzhou Institutes of Biomedicine and Health, Chinese Academy of Sciences; Guangzhou Medical University, Guangzhou, China; 2 Guangzhou Regenerative Medicine and Health Guangdong Laboratory, Guangdong Provincial Key Laboratory of Stem Cell and Regenerative Medicine, South China Institute for Stem Cell Biology and Regenerative Medicine, Hefei Institue of Stem Cell and Regenerative Medicine, Institute for Stem Cell and Regeneration, Guangzhou Institutes of Biomedicine and Health, University of Chinese Academy of Sciences, Chinese Academy of Sciences, Guangzhou, China; 3 Institute of Physical Science and Information Technology, Anhui University, Hefei, China; 4 The First Affiliated Hospital, Guangzhou Medical University, Guangzhou, China

## Abstract

Hexadimethrine bromide (Polybrene) was once used clinically as a heparin neutralizer and has recently found use as a promoter in virus-mediated gene therapy trials and gene transfer in research. However, the potential for tissue-specific toxicity of polybrene at low doses has been ignored so far. Here, we found that after intracerebroventricular (ICV) polybrene injection, mice showed disability of movement accompanied neural death and gliosis in brain, and in human neurons, polybrene induces concentration-dependent neuritic beading and fragmentation. Mechanistically, polybrene induces a rapid voltage-dependent calcium channel (VDCC)-mediated influx of extracellular Ca^2+^. The elevated cytoplasmic Ca^2+^ activates DRP1, which leads to mitochondrial fragmentation and metabolic dysfunction. At the same time, Ca^2+^ influx induces endoplasmic reticulum (ER) fragmentation and tightened associations between ER and mitochondria, which makes mitochondria prone to Ca^2+^ overloading and ensuing permeability transition. These results reveal an unexpected neuronal toxicity of polybrene, wherein Ca^2+^ influx serves as a regulator for both mitochondrial dynamics and ER–mitochondrial remodeling.

## Introduction

Polybrene, a quaternary ammonium salt, was firstly introduced into clinical practice as a heparin neutralizer^1^. Heparin was the mainstay in the treatment and prevention of thrombosis in such diverse clinical settings as venous thromboembolism, acute coronary syndrome cardiopulmonary bypass, and hemodialysis^2^, wherein polybrene was used to neutralize excessive doses of heparin. The toxicity of polybrene, that it causes life-threatening renal failure at doses >5 mg/kg, was soon discovered^3,4^, and the use of polybrene as a heparin neutralizer in clinical settings was discontinued^5^. However, polybrene at lower doses was popularly used to promote the efficiency of virus-mediated gene transfer both in vivo and in vitro^6–9^. Viruses are widely used as vectors for gene therapy clinical trials and gene transfer in researches^10–12^, and polybrene was believed to promote the binding of viruses on the cell surface by neutralizing the electrostatic repulsion between the opposing bilayers^13–16^. However, the possible tissue-specific toxicity of polybrene both in vivo and in vitro has not been systemically studied.

Toxic stresses could trigger neuritic degeneration, which is a common and early feature of disorders of the nervous system. Although the mechanisms underlying neuritic degeneration are incompletely understood, the influx of extracellular Ca^2+^ and the accumulation of intracellular Ca^2+^ have been implicated^17–19^. Subcellularly, mitochondrial Ca^2+^ was found to play a key role in excitotoxic neuronal injury^20,21^. Mitochondrial Ca^2+^ overload triggers an injury response through opening the mitochondrial permeability transition pore (mPTP), which leads to the loss of Δψ_m_^22–24^. Under apoptosis-inducing conditions, tightened endoplasmic reticulum (ER) and mitochondria connections were observed to make mitochondria prone to Ca^2+^ overloading and ensuing permeability transition^25^. We have previously reported that neuritic ER becomes fragmented and forms complexes with mitochondria, which induces IP3R-dependent mitochondrial Ca^2+^ elevation and dysfunction during neuritic degeneration^26^. Also, in Parkinson’s disease model or glutamate treatment, inhibition of ER Ca^2+^ release through Homer1 knockdown reserves mitochondrial function and reduces apoptosis^27,28^. Tethering of ER and mitochondria facilitates a variety of signaling processes including Ca^2+^ and lipid exchange^29–31^, wherein several proteins are enriched and play roles at the tether of ER and mitochondria^32–35^. However, the cellular Ca^2+^ signaling and organellar remodeling responses to neuritic toxic stresses remain unclear.

In the present study, we verified the neuritic toxicity of polybrene both in vivo and in vitro in the range of experimental concentrations. We demonstrate that polybrene induces a rapid influx of extracellular Ca^2+^, and initiates two independent mechanisms involved in neural degeneration: DRP1-dependent mitochondrial fragmentation and mitochondrial Ca^2+^ overload linked to ER–mitochondria interface remodeling.

## Results

### Polybrene induces degeneration of neurons in vivo and in vitro

**Fig. 1:**
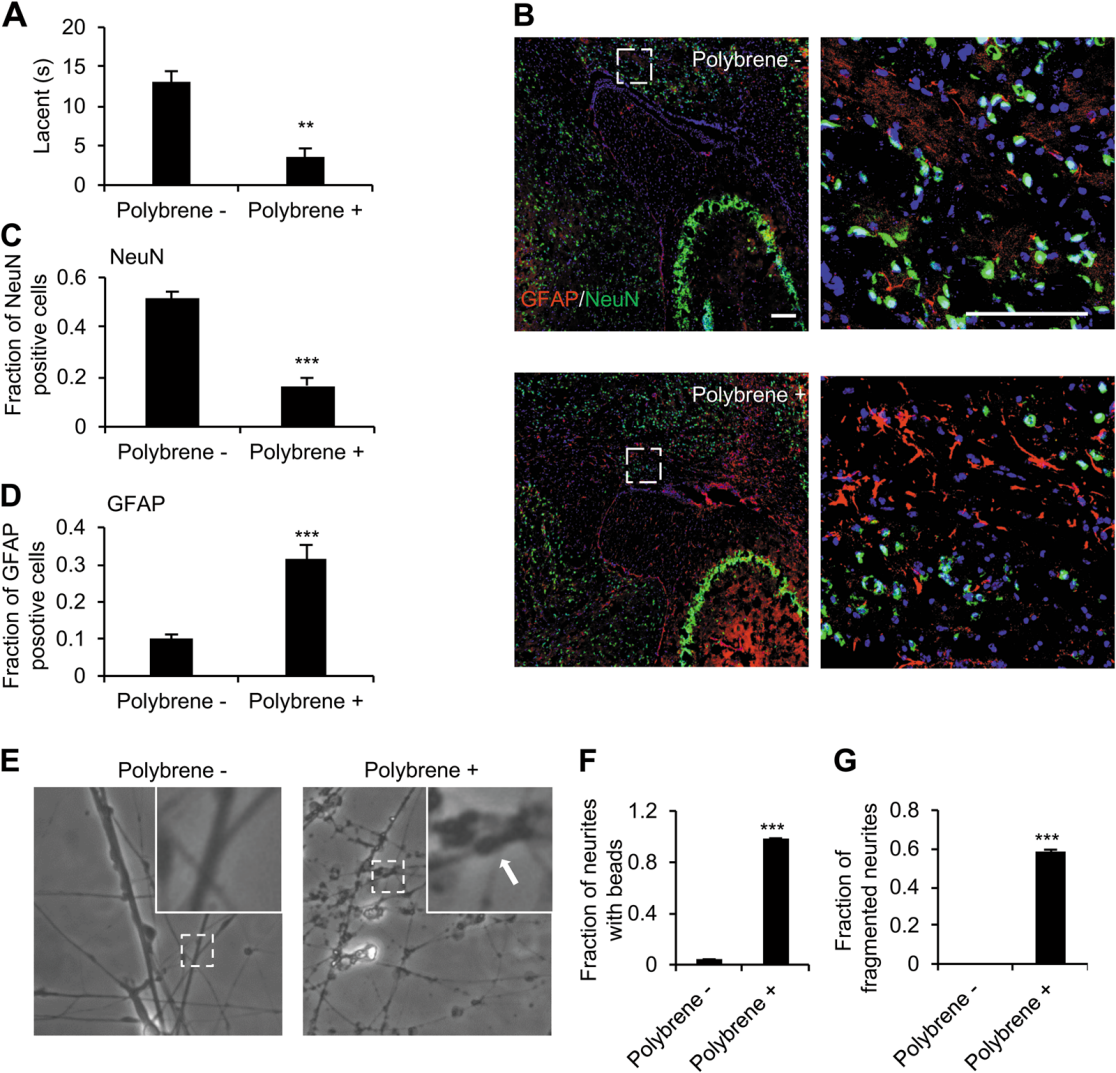
Neuritic degeneration after polybrene treatment in vivo and in vitro. **a** Performance on the accelerating rotating rod apparatus showed that the mice with polybrene injection had significantly shorter step length and less performance on the rotating rod (*n* = 4, **P< 0.01). **b–d** Altered NeuN and GFAP immunostaining after ICV injection with polybrene. **b** NeuN and GFAP immunofluorescent staining in sagittal sections from 3-month mouse brain 3 days after ICV injection of polybrene or saline. The regions enclosed by the white dotted rectangles are displayed at higher magnification at right. Quantification is shown in **c** and **d**. Scale bar: 100 μm (*n* = 4, ***P < 0.001). **e–g** Fraction of neurites with beads and fragmented neurites in neurons treated with polybrene for 24 h. The neurites with beads are indicated by arrow. Scale bar: 25 μm (*n* = 4, ***P < 0.001)

To investigate possible effects of polybrene on neurons in vivo, polybrene (4 μg/μL) or saline was intracerebroventricularly (ICV) injected to mouse brains as previously described^36^. Surprisingly, mice treated with polybrene showed disability in movement at day 3 after polybrene injection in comparison to mice treated with saline as assayed by the Rotating Rod test (Fig. 1a and SI Movie). We next performed immunostaining with antibodies specific to neuronal nuclei (NeuN), a neural marker, and glial fibrillary acidic protein (GFAP), a marker of reactive astrocytosis. Robust reduction of NeuN staining and enhancement of GFAP was observed, indicating polybrene induces neural death and gliosis (Fig. 1b–d). As increased GFAP expression in hypertrophic astrocytes often accompanies neural degeneration^37,38^, these results imply polybrene induces neural degeneration in vivo.

Then we investigated the effects of polybrene on human neurons in vitro using induced pluripotent stem cell (iPSC)-differentiated neuron system. In neurons differentiated from human iPSCs, we treated neurons with increasing concentration of polybrene (0, 0.25, 0.5, 1, 2, 4, 8, 16, and 32 μg/mL), and showed polybrene has concentration-dependent effects. As low as 4 μg/mL, obvious neuritic beads appeared, while 8 μg/mL polybrene induced neuritic fragmentation (SI Fig. 1a-c). Thus, we settled on a concentration of 8 μg/mL for further experiments, which is sufficient to induce a high fraction of neuritic beading and fragmentation (Fig. 1e–g). As reactive oxygen species (ROS) have been reported to be a key and early step in neural degeneration^39–42^, we also found ROS levels significantly increased in neurons after polybrene treatment (SI Fig. 1d, e). The neural toxicity of retroviruses, which is specific to neurons while not affecting glial cells, was reported more than 20 years ago^43^. It has been proposed that the toxicity stemmed from one or more agents in the viral medium but not retrovirus itself^43^. We systemically screened all the agents in the medium (data not shown), and found that it is, in fact, polybrene at a concentration of 8 μg/mL in retroviruses infection system that causes neural degeneration. These results demonstrate that polybrene induces degeneration of neurons both in vivo and in vitro.

### Voltage-dependent calcium channel-mediated Ca^2+^ influx is essential for polybrene-induced neural degeneration

**Fig. 2:**
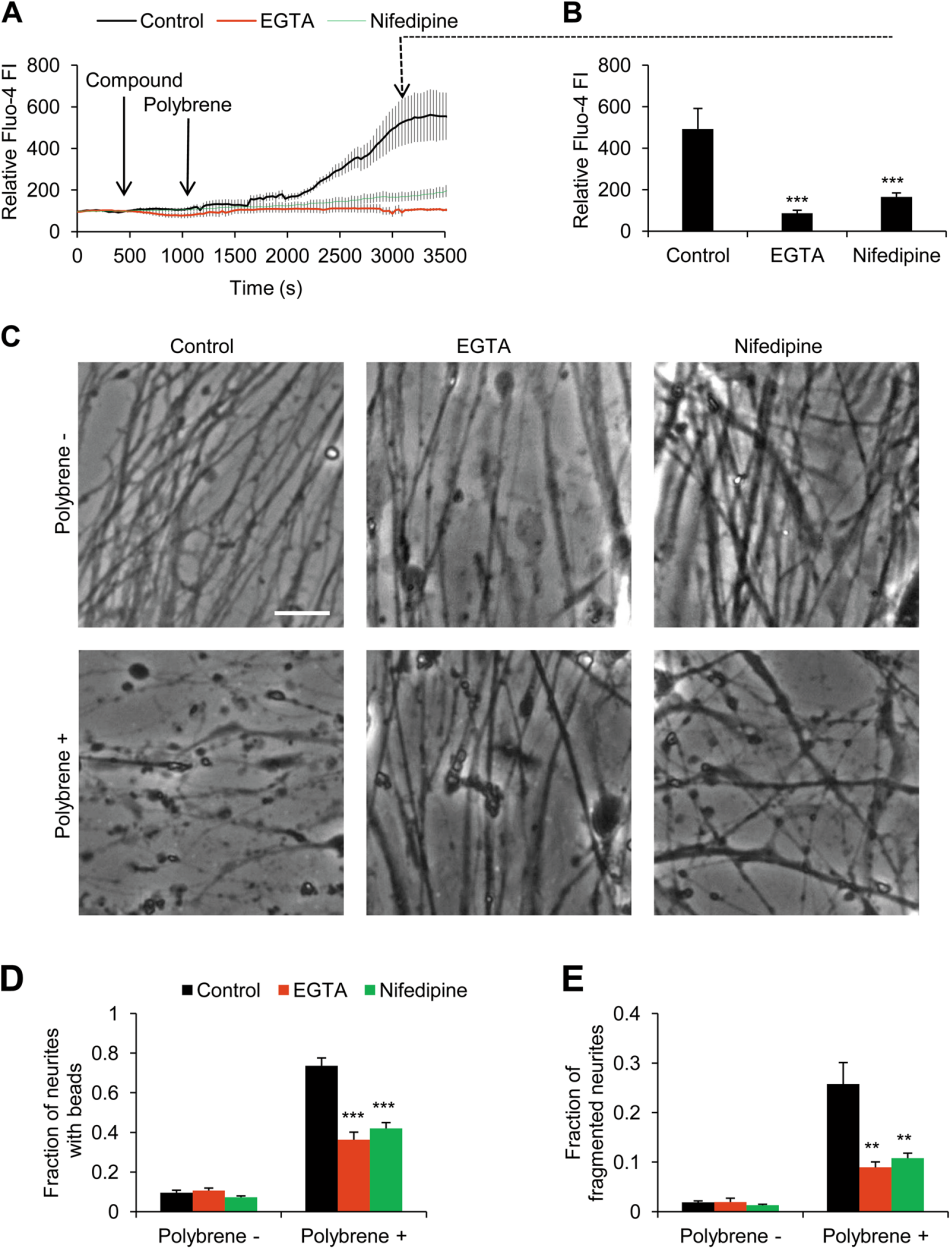
Neuritic degeneration induced by polybrene is dependent on Ca^2+^ influx. **a**, **b** Ca^2+^ influx induced by polybrene was inhibited by EGTA or nifedipine. Neurons were labeled by Fluo-4. **a** The time course of relative Fluo-4 fluorescence intensity was recorded. **b** The relative Fluo-4 fluorescence intensity at 3000 s. The fluorescence intensity of Fluo-4 at initiating time was normalized to 100 (*n* ≥ 6, ***P < 0.001). **c–e** Neuritic degeneration induced by polybrene was prevented by EGTA or nifedipine. **c** Images of neurons after polybrene treatment for 12 h with or without EGTA or nifedipine. Scale bar: 25 μm. Fractions of neurites with neuritic beads (**d**) and fragmented neurites (**e**) are shown (*n* ≥ 4, **P < 0.01, ***P < 0.001)

As Ca^2+^ signaling plays important roles in neural degeneration^44^, we checked whether polybrene stimulation affects intracellular Ca^2+^ level. Fluo-4 acetoxymethyl (Fluo-4) staining showed that polybrene triggered an increase of intracellular Ca^2+^ within 20–30 min (Fig. 2a). We next used a Ca^2+^ chelator, EGTA, together with polybrene treatment. This extracellular Ca^2+^ chelation clearly prevented neuronal bead formation and fragmentation, which demonstrates that extracellular Ca^2+^ influx mediates polybrene-induced neural degeneration (Fig. 2a–e). To determine whether Ca^2+^ influx via voltage-dependent calcium channel (VDCC) at the plasma membrane is involved in this process, we used a VDCC inhibitor, nifedipine together with polybrene treatment. Nifedipine prevented the intracellular Ca^2+^ concentration increase by polybrene and also neuronal beading and fragmentation (Fig. 2a-e). These results imply that VDCC-mediated Ca^2+^ influx is an important early cytosolic step in polybrene-induced neural degeneration.

### Ca^2+^ influx induces DRP1-dependent mitochondrial fragmentation in polybrene-induced degeneration

**Fig. 3:**
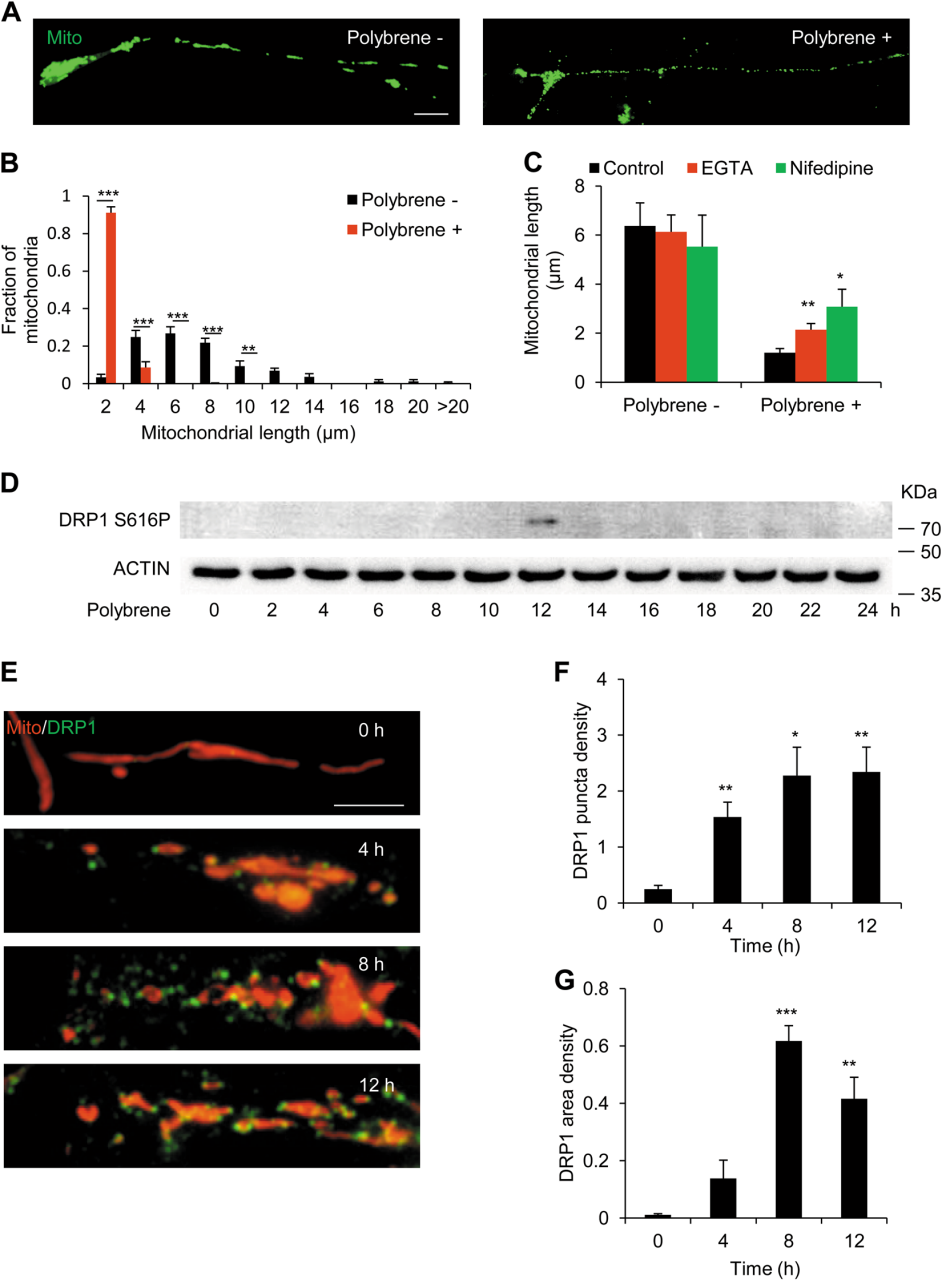
Mitochondrial fragmentation is dependent on Ca^2+^ influx in neurites treated with polybrene. **a**, **b** Mitochondrial fragmentation in neurons treated with polybrene. **a** Images of neurons expressing mito-GFP to monitor mitochondrial length after polybrene treatment for 24 h and **b** the fraction of 0–2, 2–4, 4–6, 6–8, 8–10, 10–12, 12–14, 14–16, 16–18, 18–20, and > 20 μm mitochondria of total mitochondria in neurites treated with polybrene for 24 h versus control. Scale bar: 10 μm (*n* ≥ 5, **P < 0.01, ***P < 0.001). **c** EGTA or nifedipine inhibits mitochondrial fragmentation induced by polybrene. Quantification the average mitochondrial length in neurons treated with polybrene and EGTA or nifedipine for 12 h (*n* = 4, *P < 0.05, **P < 0.01). **d** Western blot shows DRP1 S616P level in neurons during the time course treated with polybrene. **e–g** DRP1 localizes to mitochondria in neurites treated with polybrene. **e** The immunofluorescent staining of DRP1 in neurons expressing mito-DsRed after polybrene treatment for 0, 4, 8, 12 h. Scale bar: 10 μm. **f** DRP1 puncta density (number of puncta/mitochondrial area). **g** DRP1 area density (area of puncta/mitochondrial area) (*n* ≥ 5, **P < 0.01, ***P < 0.001)

As one target of cytosolic Ca^2+^ increase is DRP1 phosphorylation-mediated mitochondrial fragmentation^45,46^, we next monitored mitochondrial morphology after polybrene treatment. We found that mitochondria were significantly shorter after polybrene treatment, with most lengths less than 2 μm (Fig. 3a, b). To investigate the role of extracellular Ca^2+^ influx in mitochondrial fragmentation, we added EGTA and nifedipine in polybrene treatment. Either EGTA or nifedipine inhibited mitochondrial fragmentation induced by polybrene (Fig. 3c). These results indicate the mitochondrial fragmentation is a consequential event after the intracellular Ca^2+^ increase induced by polybrene.

Mechanistically, we found that DRP1 phosphorylated at S616, the active scission form, could be detected only at 12 h during 24-h polybrene treatment (Fig. 3d). Since DRP1 activation leads to its recruitment to the surface of the mitochondria^47^, we also performed immunostaining to detect DRP1 puncta on mitochondria. We quantified the number of DRP1 puncta recruited to mitochondria as the puncta density (number of puncta/mitochondrial area) and DRP1 area density (area of puncta/mitochondrial area), both of which increased after polybrene treatment (Fig. 3e). Furthermore, we showed that either EGTA or nifedipine decreased the puncta density and area density of DRP1 after polybrene treatment (SI Fig. 2b-d), demonstrating that this mitochondrial DRP1 recruitment is Ca^2+^ influx dependent. Finally, we showed that DRP1-K38A, a dominant-negative mutant of DRP1^48,49^, could rescue polybrene-induced mitochondrial fragmentation (SI Fig. 3a, b). In addition, we detected the protein expressions of other fission proteins, e.g., MFF and FIS1, and did not observe upregulation of either. This indicates that mitochondrial fragmentation induced by polybrene is not dependent on MFF and FIS1 (SI Fig. 2a). Collectively, our results imply that Ca^2+^ influx induces DRP1-dependent mitochondrial fragmentation in polybrene-induced neural degeneration.

### DRP1-K38A rescues metabolic dysfunction and neuritic degeneration induced by polybrene

**Fig. 4:**
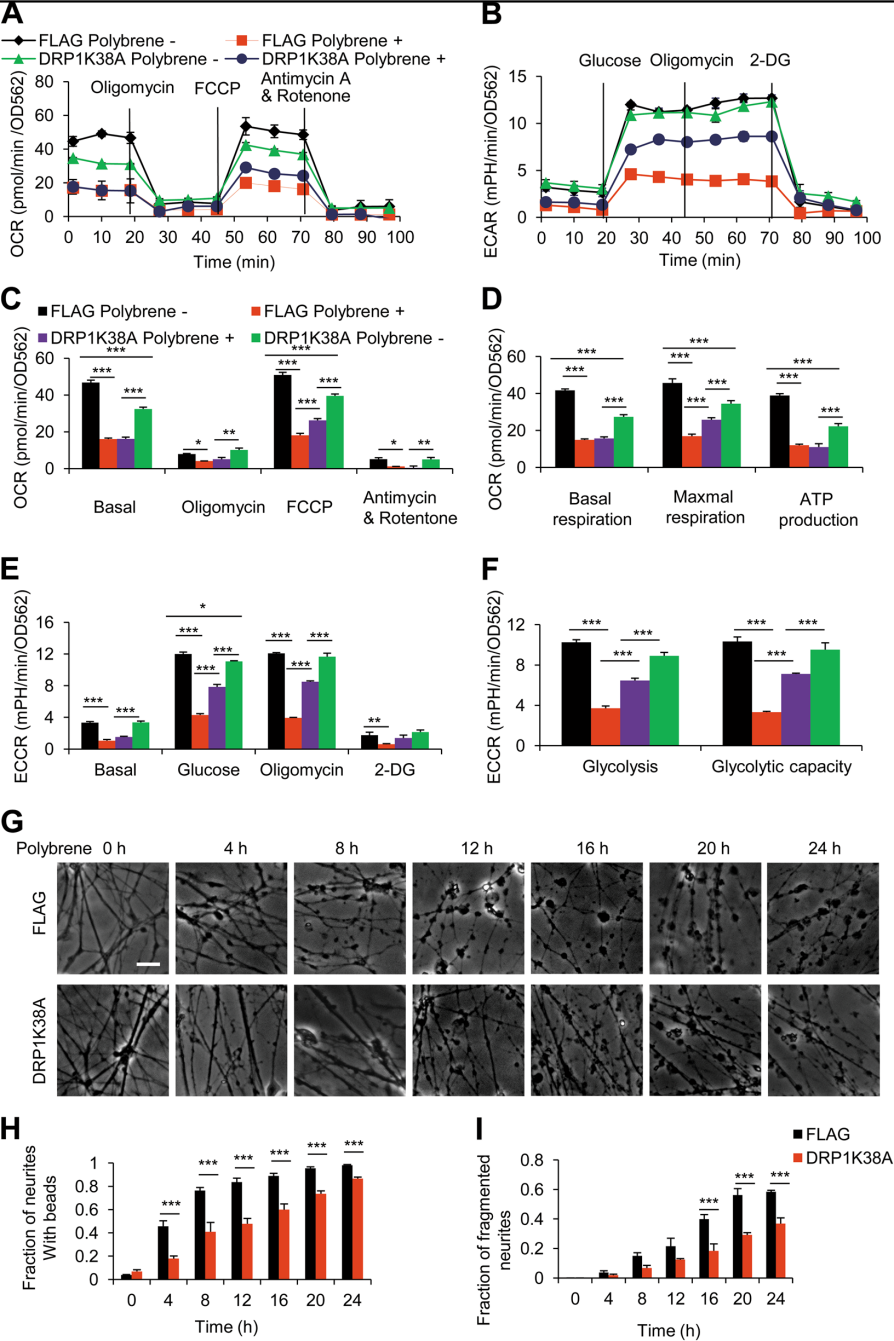
DRP1-K38A prevents metabolic dysfunction and neural degeneration induced by polybrene. **a–f** DRP1-K38A prevents metabolic dysfunction induced by polybrene. Neurons were infected with DRP1-K38A viruses or FLAG as control, then treated with or without polybrene for 12 h. **a** Oligomycin (which inhibits ATP synthesis), FCCP (which induces maximum respiratory capacity), and rotenone (which inhibits total mitochondrial respiration) were added sequentially, and the OCR, indicative of oxidative phosphorylation, was measured in real time. **b** Glucose, oligomycin (which inhibits ATP synthesis), and 2-DG (which inhibits total glycolysis) were added sequentially, and the ECAR, indicative of extracellular acidification rate, was measured in real time. **c** Quantification of the OCR for each cellular stressor in **a**. **d** Basal respiration (subtraction of the basal OCR from the antimycin A and rotentone OCR), maximal respiration (subtraction of the FCCP OCR from the antimycin A and rotentone OCR), and mitochondrial ATP production capacity (subtraction of the oligomycin OCR from the basal OCR). **e** Quantification of the ECAR for each cellular stressor in **b**. **f** Glycolysis (subtraction of the basal ECAR from the glucose ECAR) and glycolytic capacity (subtraction of the basal ECAR from the oligomycin ECAR) (*n* ≥ 3, *P < 0.05, **P < 0.01, ***P < 0.001). **g–i** Neurons expressing FLAG or DRP1-K38A at 0, 4, 8, 12, 16, 20, 24 h time course after polybrene treatment (**g**). Scale bar: 25 μm. **h** Fraction of neurites with neuritic beads in **g**. **i** Fraction of fragmented neurites in **g** (*n* ≥ 3, ***P < 0.001)

To investigate the possible metabolic dysfunction mediated by DRP1 in polybrene-induced neural degeneration, we detected the effect of DRP1-K38A on cellular metabolism using a Seahorse Flux Analyzer to measure the mitochondrial oxygen consumption rate (OCR) and the extracellular acidification rate (ECAR). Polybrene treatment caused defects in both mitochondrial OXPHOS (basal respiration, maximal respiration, and mitochondrial ATP production) and glycolysis (glycolysis and glycolytic capacity), whereas DRP1-K38A partly restored the maximal respiration and glycolysis (Fig. 4a–f).

Then we asked whether DRP1-K38A could rescue Ca^2+^ influx-mediated neural degeneration induced by polybrene. We detected the time course of neuritic degeneration in DRP1-K38A expressing neurons after polybrene treatment, and showed that the fraction of neurites with beads and fragmentation decreased in comparison with that in control (Fig. 4g–i). Then we investigated the relationship between extracellular Ca^2+^ influx and DRP1 in degenerated neurons induced by polybrene, and found DRP1-K38A had no further inhibitory effect either in EGTA or nifedipine treatment group (SI Fig. 3c, d). This verifies that DRP1-K38A mutant and inhibition of extracellular Ca^2+^ influx functions in the same pathway. Together, our results imply that DRP1 inhibition could rescue metabolic dysfunction and neuritic degeneration in neurons treated with polybrene.

### Mitochondrial Ca^2+^ overload and ER–mitochondria complex formation in polybrene-induced degeneration

**Fig. 5:**
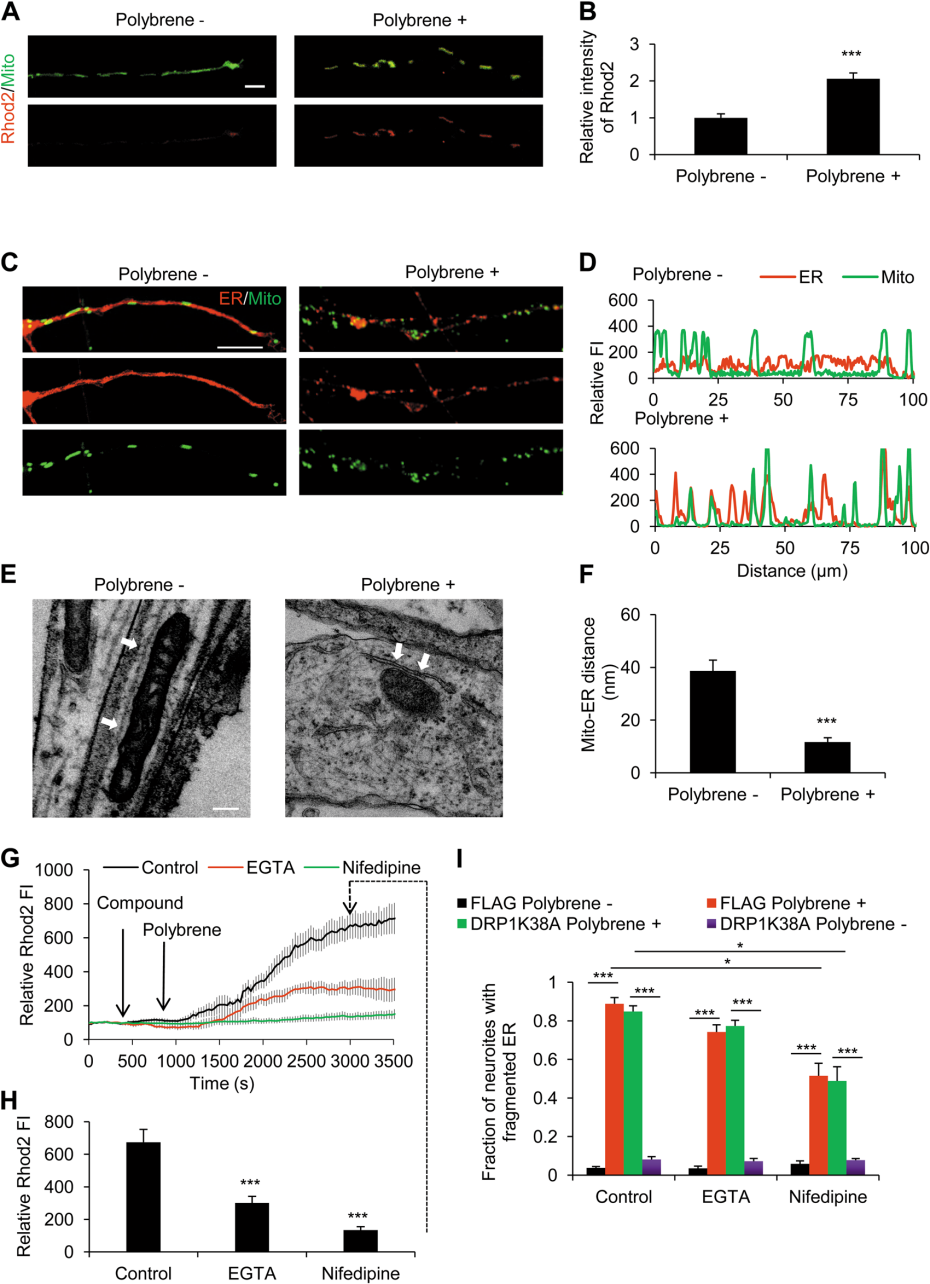
Mitochondrial Ca^2+^ overload and ER–mitochondria complex formation in neurons treated with polybrene. **a**, **b** Mitochondrial Ca^2+^ concentration increases during neuritic degeneration induced by polybrene: **a** Images of neurons stained with Rhod2 to monitor neuritic mitochondrial Ca^2+^ concentration after polybrene treatment for 12 h and **b** quantification of the Rhod2 fluorescence intensity. Fluorescence intensity of control was normalized to 1. Scale bar: 10 μm (*n* ≥ 15, ***P < 0.001). **c**, **d** Distal neuritic ER becomes fragmented and co-localizes with mitochondria in neurons treated with polybrene for 24 h. **c** Neurons were infected with ER-DsRed and mito-GFP viruses after differentiation. In the image panels, the top panel is a merge of the two lower panels. The left panels indicate controls, and the right panels indicate polybrene treatment. Scale bar: 10 μm. Analysis of fluorescence intensity performed on neurites presented in **d** using the ZEN software from Zeiss. Fluorescence intensity profiles of ER and mitochondria are plotted from soma to distal direction along neurites. **e**, **f** TEM analysis of ER in neurites treated with polybrene for 24 h. ER profiles are indicated by arrows. Scale bar: 500 nm. **f** Qualification of the distance of ER /mitochondria in neurites (*n* ≥ 9, ***P < 0.001). **g**, **h** The effect of EGTA or nifedipine on mitochondrial Ca^2+^ concentration increase induced by polybrene. **g** Neurons were labeled by Rhod2 to monitor mitochondrial Ca^2+^ concentration. **h** The relative Rhod2 fluorescence intensity at 3000 s is shown. The fluorescence intensity of Rhod2 at initiating time was normalized to 100, respectively (*n* ≥ 6, ***P < 0.001). **i** The effect of EGTA or nifedipine on neuritic ER fragmentation induced by polybrene. Neurons were treated with polybrene simultaneously with or without EGTA or nifedipine for 12 h. The fraction of neurons with fragmented neuritic ER was calculated (*n* = 3, *P < 0.05)

As mitochondrial Ca^2+^ signaling plays an important role in neural degeneration^50^, we asked whether polybrene inducing Ca^2+^ influx results in mitochondrial Ca^2+^ elevation. We conducted imaging of mitochondrial Ca^2+^ by Rhod-2-acetyl ester (Rhod2), and showed that the neuritic mitochondrial Ca^2+^ concentration significantly increased after polybrene treatment (Fig. 5a, b). Considering mitochondrial Ca^2+^ overload causes mitochondrial dysfunction^22,23^, we tested parameters such as mPTP state, ΔΨ_m_, and mitochondrial mobility. We performed calcein acetoxymethyl ester (calcein) assay in the presence of CoCl_2_ to monitor mPTP state, a procedure by which the calcein signal was quenched by Co^2+^ in the cytosol or in mitochondria that have undergone permeability transition. The results showed that mPTP was opened after polybrene treatment, compared with a closed mPTP state in control (SI Fig. 4a, b). We then used the far-red fluorescent dye 1,1′,3,3,3′,3′-hexamethylindodicarbocyanine iodide (Dilc(5)) to monitor ΔΨ_m_ and showed ΔΨ_m_ dissipation induced by polybrene treatment (SI Fig. 4c, d). By quantifying mitochondrial motility, we found mitochondrial motility is completed eliminated in neurons treated with polybrene (SI Fig. 4e-g).

We previously reported that ER fragments and forms complexes with mitochondria during degeneration induced by axotomy, which is the physical cause of mitochondrial Ca^2+^ overload^26^. Thus, we detected the morphology of ER and ER–mitochondria complexes under polybrene treatment. Neurons were infected with ER-DsRed and mito-GFP lentiviruses to mark ER and mitochondria, respectively. After polybrene treatment, neuritic ER becomes fragmented and forms complexes with mitochondria. Fluorescence profile analysis showed the co-localization of fragmented ER and mitochondria (Fig. 5c, d). We also detected the morphology of ER and mitochondria in gliacytes derived from iPSCs, and found there is no obvious change after polybrene treatment (SI Fig. 5). These results demonstrated that gliacytes are not susceptive to polybrene. Neuritic ER–mitochondria complexes were also verified by electron microscopy, which showed the contact between ER and mitochondria was much narrower in neurites after polybrene treatment (11.65 ± 1.67 nm) than in controls (38.71 ± 3.98 nm) (Fig. 5e, f). Together, our data suggest that mitochondrial Ca^2+^ overload by the tightened ER–mitochondrial coupling and consequent mitochondrial dysfunction are involved in polybrene-induced neuritic degeneration.

Then, we asked how extracellular Ca^2+^ influx is involved in ER–mitochondria complex and mitochondrial Ca^2+^ elevation. We found that either EGTA or nifedipine inhibits ER fragmentation, though nifedipine had a more significant inhibitory effect. When we detected the effect of EGTA or nifedipine on mitochondrial Ca^2+^ accumulation, nifedipine also more significantly inhibits the increase of mitochondrial Ca^2+^ concentration induced by polybrene (Fig. 5g, h).

Our above results demonstrate the role of DRP1 in polybrene-induced mitochondrial fission and metabolic defects. We then tested whether DRP1 is also involved in ER–mitochondria complex formation and mitochondrial Ca^2+^ elevation. We found that DRP1-K38A could not inhibit ER fragmentation induced by polybrene and could not enhance the inhibition of either EGTA or nifedipine (Fig. 5i). These results indicate two mitochondrial pathways in neuritic degeneration induced by polybrene, ER–mitochondria complex/mitochondrial Ca^2+^ elevation and mitochondrial fission/metabolic defects, are distinct.

## Discussion

Polybrene was first widely used at high doses as an anti-heparin drug in the clinic^1^, but its use was soon discontinued because of its renal toxicity^3,4^. Polybrene has also been widely used as a virus infection promoting agent in gene therapy and laboratory gene transfer, typically in the concentration range of 4 μg/mL–8 mg/mL. However, the possible toxicity of polybrene at lower dose has not been investigated. Our results for the first time indicate neural toxicity of polybrene in a concentration-dependent manner. Doses as low as 4 μg/mL cause neural degeneration, which would potentially limit its clinical use. Besides its potential danger for neurons in gene therapy, the presence of polybrene also seriously interferes with the results in various neural research systems.

As other heparin neutralizer drugs have found wide use in the clinic such as protamine^2^. Protamine, which appears to function in a similar manner of polybrene, might also be an alternative to polybrene in retroviral-mediated human gene therapy^51,52^. Regardless, our work suggests that their safety in clinical usage should be re-evaluated, especially for their neural toxicity.

In neurons, the fission/fusion machinery proteins maintain mitochondrial integrity. The fission proteins include DRP1, MFF, and FIS1, and the fusion proteins include MFN1/2 and OPA1^49,53^^,^^54^. Our results showed that DRP1 is activated, while either expression of MFF and FIS1 does not increase after polybrene treatment. Overexpression of DRP1 dominant-negative mutant protein DRP1-K38A could rescue polybrene-induced mitochondrial dysfunction, which indicates that DRP1-dependent mitochondrial fission is abnormally activated by polybrene treatment. We further detected the expression of fusion proteins, and the protein levels of MFN1/2 and OPA1 do not decrease after polybrene treatment (data not shown), ruling out the possibility of fusion activity reduction association with mitochondrial fragmentation in this process.

The increase of intracellular Ca^2+^ concentration under neuropathological conditions is caused by the release of Ca^2+^ pool such as ER, or the extracellular Ca^2+^ influx through Ca^2+^ channels across the plasma membrane^55,56^. Here, we demonstrate that extracellular Ca^2+^ influx via VDCC causes intracellular Ca^2+^ concentration increase and subsequent effects. Homer1, a postsynaptic scaffolding protein regulating intracellular calcium mobilization, has been reported to play a role in both glutamate-mediated excitotoxicity and neuronal injury in in vitro Parkinson’s disease model^27,28^. In former model, Homer1 knockdown protects neurons partially on the inhibition of Ca^2+^-dependent ROS production and the preservation of the ER and mitochondrial function^27,57^. In latter model, Homer1 knockdown has protective effects by reducing Ca^2+^ overload-mediated ROS generation, and partially on the regulatory effects on Ca^2+^ channels in both plasma membrane and ER^28^. Thus, Homer1 may play common roles in drug-induced neural injury, and has possibility to be involved in polybrene treatment.

**Fig. 6:**
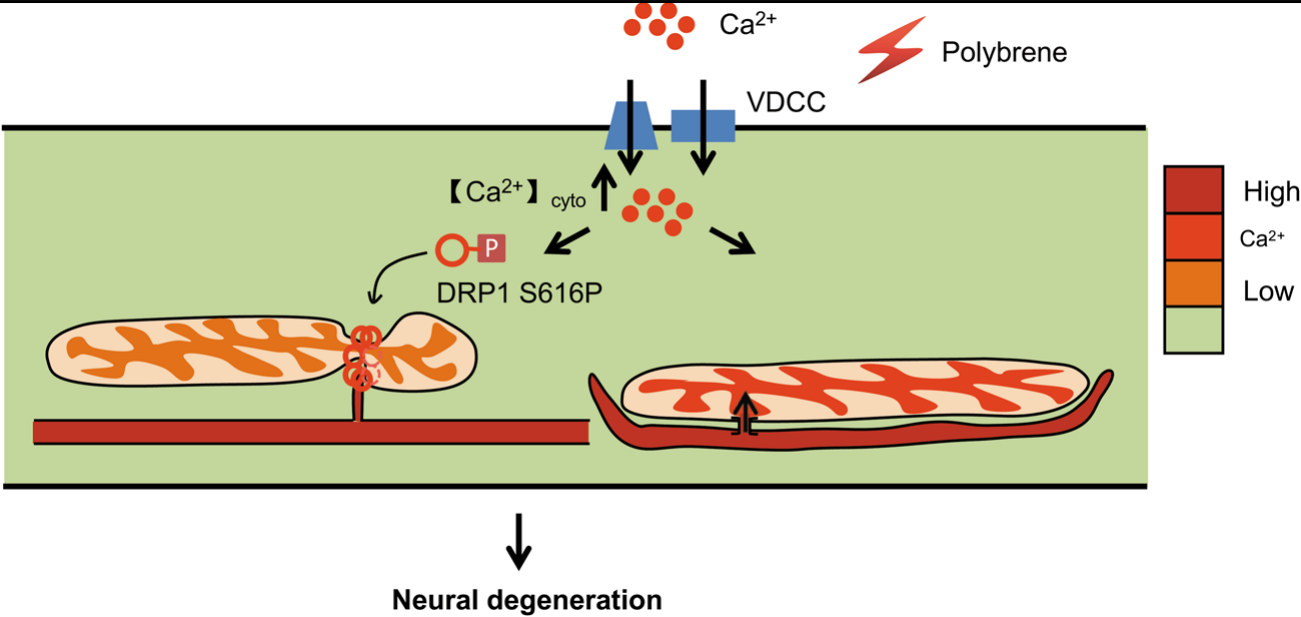
Model of neuritic degeneration induced by polybrene. In response to polybrene, a rapid extracellular Ca^2+^ influx occurs, which initiates two independent mechanisms involved in neural degeneration: DRP1-dependent mitochondrial fragmentation and ER-mitochondria interface remodeling

Our findings also shed light on the pathological mechanisms of drug-induced neuritic degeneration (Fig. 6). In response to polybrene, a rapid extracellular Ca^2+^ influx occurs, which causes intracellular Ca^2+^ concentration increase and triggers two independent mechanisms: DRP1 activation and ER–mitochondria complex formation. On the one hand, the activated DRP1 is recruited to mitochondria, which leads to mitochondrial fragmentation and subsequent metabolic defects. On the other, neuritic ER becomes fragmented and forms complexes with mitochondria, which causes mitochondrial Ca^2+^ overload and dysfunction, consistent with our previous report in an axotomy model^26^. We demonstrate that ER fragmentation is determined by extracellular Ca^2+^ influx via VDCC, but more work needs to be done to explore the mechanism in future.

## Methods

### Ethical statement

The experiments involving human subject and animal research were reviewed and approved by the Guangzhou Institutes of Biomedicine and Health Ethical Committee.

### Animal studies

Adult (2–3 months) BALB/c mice obtained from our in-house breeding colony were used in all experiments. ICV cannulation was performed under aseptic conditions as described previously^58^. In brief, 4 μg/μL polybrene was incubated at 37 °C for 15 min, and ICV injection of 5 μL mixture was performed for ≥20 min.

At day 3 after ICV injection, Motor Performance Test-Accelerating Rotating Rod Test was performed. The rotating rod apparatus was used to evaluate motor function. The mice were placed on the rod (3 cm in diameter) for four trials. Each trial lasted for a maximum of 5 min, during which the rotating rod underwent linear acceleration from 0 to 30 rpm and then remained at maximum speed. Animals were scored for their latency to fall (in seconds) in each trial.

Then the mice were deeply anesthetized and euthanized by transcardiac perfusion with fixative solution (4% paraformaldehyde and 2% glutaraldehyde in 0.1 M sodium cacodylate buffer, pH 7.4, 50 mL/animal). The brains were harvested and postfixed in 4% paraformaldehyde, dehydrated in increasing concentrations of sucrose (30–60%), infiltrated in OCT Tissue Freezing Medium (380148, Leica), and plastic-embedded. Frozen sections (15 μm) were cut, mounted on coverslips, and then dried at room temperature and stained with NeuN (ab128886, Abcam, 1:500) and GFAP (Z033429, Dako, 1:1000).

### Plasmids and lentivirus production

Mito-GFP, mito-DsRed, ER-DsRed, DRP1-K38A plasmids were subcloned into a lentiviral expression vector—pRlenti.

HEK 293T cells were used for lentivirus production, which were grown in Dulbecco’s modified Eagles medium (DMEM) supplemented with 10% fetal bovine serum, streptomycin (50 μg/mL) and penicillin (50 U/mL). All cultures were maintained in a humidified incubator containing 5% CO_2_ at 37 °C. HEK 293T cells were plated onto 10-cm dishes and co-transfected using the Ca^2+^ phosphate method with target plasmids and packaging vectors PMD2.G, PSPAX2. The viruses were harvested by centrifuging at 50,000×*g* for 2.5 h, and then used to infect cells.

### iPSC culture, neuron differentiation, virus infection, and polybrene treatment

Normal human iPSCs were derived from ATCC IMR90 cells^59^. Human iPSCs were cultured in mTesR1 medium (05857, Stem Cell). All iPSC colonies were transferred by EDTA onto Matrigel (356234, Becton Dickinson). Human iPSCs were differentiated to neurons according to the protocol described previously^26^. Briefly, iPSCs were derived for embryoid bodies (EBs) formation, and after 4 days, the EB medium was changed to N2 medium for 3 days. Then EBs were attached to Matrigel for another 7 days in N2 medium. Then neural rosettes were detached and cultured in suspension in N2B27 medium to form neurospheres. For neuron and gliocyte differentiation, neurospheres (20–40 neurospheres per well of 6-well dishes) were plated onto matrigel-coated glass coverslips and cultured in N2B27 medium supplemented with 10 ng/mL BDNF (4004, BioVision) and 1 μM cAMP (A6885, Sigma). After 4–6 days of differentiation, lentiviruses were used to infect cells without polybrene. Three days after infection, neurons and gliocytes derived from neurospheres were treated with polybrene.

### Assessment of beading and fragmented neurites

The method for assessment of beading and fragmented neurites is the same as in our previous report^26^. Briefly, neurons were treated with polybrene for 24 h. For the polybrene treatment with 5 mM EGTA or 50 µM nifedipine (S1808, Selleck), drugs were added simultaneously with polybrene for 12 h. Neurons were observed under a phase-contrast microscope. More than 100 neurons in duplicate wells were assessed blindly in three independent trials. The ratio of neurites with neuritic beads or fragmented neurites was calculated as a percentage of total neurites.

### Measurement of intracellular Ca^2+^ and mitochondrial Ca^2+^

Fluo-4 (F-14201, Invitrogen) was used to measure intracellular Ca^2+^. Cells were incubated with 1 µM Fluo-4 for 30 min at 37 °C, and then washed twice with medium before imaging.

Rhod2 (R-1244, Invitrogen), an indicator of mitochondrial Ca^2+^, was used to measure mitochondrial Ca^2+^. Cells were first plated on glass coverslips, and then incubated with 1 µM Rhod2 for 30 min at 37 °C. Cells were further incubated for 30 min after being washed in indicator-free medium. After loading Rhod2, cells were returned to growth conditions for an additional 18 h to eliminate the residual cytosolic fraction of the Rhod2 probe. The fluorescence was detected by Zeiss confocal microscope. Neuritic mitochondrial Rhod2 fluorescence intensity was analyzed by the method previously reported with minor modification^60,61^.

For the time course experiments of Fluo-4 or Rhod2, neurons were loaded with Fluo-4 or Rhod2, and the fluorescence intensity of Fluo-4 or Rhod2 was recorded for 5 min; EGTA, nifedipine, or water was added for additional 5 min. then polybrene was added and recorded for 50 min.

### Measurement of neuritic mitochondrial length

For measuring neuritic mitochondrial length, mito-DsRed lentivirus was used to mark neuritic mitochondria, and micrographs were taken with a Zeiss confocal microscope. ImageJ software was used to quantify the length of neuritic mitochondria. Mitochondrial length was calculated by tracing the individual neurites in more than 10 separate neurons taken randomly for each treatment (from three separate experiments).

### Western blot analyses

The neuron cells were lysed in RIPA buffer (P0013K, Beyotime) containing both phosphatase inhibitor cocktail and protease inhibitor cocktail. Total proteins were electroporesed on 12% polyacrylamide gel containing sodium dodecyl sulfate and immediately transferred onto the PVDF membrane (ISEQ00010, Millipore). Blots were incubated overnight at 4 °C with the appropriate primary antibodies, followed by incubation with secondary antibody for 1 h at room temperature, and then visualized using ECL substrate solution (P0018, Beyotime). The following primary antibodies were used: Phospho-DRP1 (Ser616) (DRP1S616P) (3455, Cell Signal, 1:1000), FIS1 (10956-1-AP, Proteintech, 1:1000), MFF (1 7090-1-AP, Proteintech, 1:1000), and ACTIN (A2103, Sigma, 1:5000).

### Immunofluorescence

Cells were fixed with 4% paraformaldehyde (pH 7.4) for 30 min at room temperature. The cells were incubated with primary antibodies in PBS containing 10% goat serum, 0.3% Triton X-100 overnight at 4 °C, washed and then incubated with secondary antibodies for 1 h. Fluorescence images were acquired on Zeiss confocal microscope. The primary antibody was DRP1 (ab184247, Abcam, 1:500).

Mitochondrial DRP1 puncta were analyzed by the method previously reported with minor modification^62^. Briefly, a binary mask of the mitochondrial channel mito-DsRed was created for subtracting all extra-mitochondrial DRP1 fluorescence. To select mitochondrial DRP1 puncta for analysis, mitochondrial DRP1 fluorescence was thresholded. The thresholding value was determined as the average threshold value needed to select mitochondrial DRP1 puncta in control. To count mitochondrial puncta and measure their area, the thresholded image was converted to a binary image. The ratio of DRP1 puncta number to mitochondrial area indicates DRP1 puncta density, and the ratio of DRP1 puncta area to mitochondrial area indicates DRP1 area density.

### OCR and ECAR measurements using Seahorse Cellular Flux assays

Seahorse plates were pre-treated by coating with 0.1 mg/mL poly-d-lysine and Matrigel thereafter. Neural stem cells were passaged and seeded in neural medium for neural differentiation onto pre-treated Seahorse plates with 5 × 10^5^ per XF24 well to ensure about 90% surface coverage at the time of the experiment. After 7 days of differentiation, FLAG or DRP1-K38A virus was used to infect the neurons. Infected neurons were cultured for 2 weeks and treated with polybrene for 12 h before the measurement of OCR and ECAR. Medium was exchanged for glycosis stress medium (XF Base medium (102353, Seahorse Bioscience) supplemented with 2 mM glutamine) 1 h before the assay and kept for the duration of the measurement. Substrates and selective inhibitors were injected during the measurements to achieve final concentrations of glucose (2.5 mM), oligomycin (1 μM), 2-DG (100 mM), FCCP (0.5 μM), and rotenone/antimycin A (1.0 µM) according to the manufacturer’s instructions. The OCR and ECAR values were further normalized to OD562 (BCA protein assay). The baseline of OCR and ECAR were defined as the average values. Changes in OCR and ECAR in response to substrates and inhibitors addition were defined as the maximal change after the chemical addition compared with the baseline.

### Assessment of ER fragmentation and profile analysis

To assess neuritic ER fragmentation, ER-DsRed was used to mark neuritic ER. More than 100 neurites in duplicate wells were assessed blindly in four independent trials. The ratio of neurons with fragmented neuritic ER was calculated as a percentage of total neurites.

For analyzing the co-localization of ER and mitochondria, ER and mitochondria were marked by ER-DsRed and mito-GFP viruses, respectively. After polybrene treatment for 24 h, the fluorescence was detected and analyzed by Zeiss confocal microscope. The profiles of ER and mitochondria were analyzed by ZEN software.

### Transmission electron microscopy (TEM) analysis

Neurospheres were plated onto matrigel-coated glass coverslips to neural induction. Neurons were treated with polybrene for 24 h, and then cell slices were fixed in 1.5% paraformaldehyde and 1.5% glutaraldehyde in 0.1 M Sörensen’s phosphate buffer (0.1 M NaH_2_PO_4_/Na_2_HPO_4_, pH 7.2) for 1 h followed by washing three times in Sörensen’s phosphate buffer. Cells were postfixed in 1% osmium tetroxide in Sörensen’s phosphate buffer for 1 h, washed three times, and dehydrated in ethanol with increasing concentration: 25%, 50%, 75%, and 96% for 2 × 10 min, respectively, and 100% for 2 × 15 min. Prior to embedding, the slices were placed in 100% acetone for 2 × 20 min and then in a mixture of acetone and epon resin polybed 812 (Polysciences, 1:1) overnight. The specimen was transferred to pure resin for at least 4 h before embedding in new pure resin and polymerization at 60 °C for 48 h. Then embedded specimen was sectioned in an ultratome (Super Nova) at 50 nm and mounted on slot copper grids previously covered with a thin film of pioloform. Grids were stained in 4% uranyl acetate for 30 min at 40 °C and 0.5% lead citrate for 2 min at room temperature and observed with a Philips CM 10 electron microscope. Sections from four control and four polybrene-treated slices were analyzed.

### Measurement of mPTP opening, ΔΨ_m_, and ROS

For measuring mPTP opening, cells were washed twice with Hank’s buffer, and then stained with 1 µg/µL calcein (C3100MP, Invitrogen) in the presence of 1 µM CoCl_2_ for 25 min at 37 °C, and washed twice with Hank’s buffer before imaging. For measuring ΔΨm, cells were stained with 50 nM Dilc(5) (M34151, Molecular Probes) for 25 min in 37 °C, and then washed twice with serum-free medium before imaging. For measuring ROS levels, cells were incubated with 1 µM carboxy-DCFH-DA (DCFH-DA) (S0033, Beyotime) in serum-free medium for 25 min at 37 °C. The fluorescence was detected by Zeiss confocal microscope. The fluorescence intensities were analyzed as in our previous report^26^.

### Measurement of mitochondrial motility

mito-DsRed or mito-GFP was used to mark mitochondria. Neurons were transferred to Nunc™ Glass Bottom Dishes and treated with polybrene for 24 h, and then mitochondrial movements were recorded for 10 min at 4/s using Zeiss confocal microscope maintained at 37 °C.

Multiple Kymograph measurement was analyzed as in our previous report^26^. Mitochondria were subsequently classified as motile (velocity > 0.1 μm/s) or stationary (velocity < 0.1 μm/s).

### Statistics

Graph Pad Prism was used to calculate the standard error of the mean (SEM) between experimental samples. When two independent variables were being analyzed, 2-way ANOVA with Bonferroni post-analysis was performed. In all other instances, statistical differences between groups were calculated using Student’s *t* test where a *P* value of <0.05 was considered significant.

## Electronic supplementary material

Supplementary Figure legends

Supplementary Figure 1

Supplementary Figure 2

Supplementary Figure 3

Supplementary Figure 4

Supplementary Figure 5
