# Supplementary material for: Polybrene induces neural degeneration by bidirectional Ca2+ influx-dependent mitochondrial and ER–mitochondrial dynamics
Source: Cell Death Dis. 2018 Sep 20;9(10):966. doi: 10.1038/s41419-018-1009-8 (PMC6148003; doi:10.1038/s41419-018-1009-8)
Supplement: Supplementary file 1 — Supplementary Figure legends [file 41419_2018_1009_MOESM1_ESM.docx]

**SI Movie: Tracking movement activity of the mouse with polybrene ICV injection.**

**SI Fig. 1: Neuritic degeneration in neurons treated with polybrene across a range of concentrations.**

**a** Images of neurons after treatment with polybrene in concentration gradient (0, 0.25, 0.5, 1, 2, 4, 8, 16 and 32 μg/mL) for 24 h. Scale bar: 25 μm.

**b** Fraction of neurons with neuritic beads in (a) (n=3, ***, P<0.001).

**c** Fraction of fragmented neurites in (a) (n=3, **, P<0.01, ***, P<0.001).

**d, e** ROS levels increased after polybrene treatment. (**d**) Neurons were stained with DCF-DA to monitor ROS level. Scale bar: 10 μm (**e**) Quantification of the DCF-DA fluorescence intensity (n=4, *, P<0.05).

**SI Fig. 2: EGTA or nifedipine inhibits the activation of DRP1 in neurons treated with polybrene.**

**a** Western blot shows MFF and FIS1 level in neurons during the time course treated with polybrene.

**b-d** EGTA or nifedipine inhibited the localization of DRP1 to mitochondria induced by polybrene. (**b**) Immunofluorescent staining of DRP1 in neurons after polybrene treatment for 12 h simultaneously with or without EGTA or nifedipine. Scale bar: 10 μm. (**c**) DRP1 puncta density. (**d**) DRP1 area density (n≥6, *, P < 0.05,**, P < 0.01,***, P < 0.001).

**SI Fig. 3: DRP1-K38A prevents mitochondrial fragmentation induced by polybrene.**

**a** Images of neurons expressing mito-DsRed and FLAG or DRP1-K38A after 0, 4, and 8 h of polybrene treatment. Scale bar: 10 μm.

**b** Quantification of the average mitochondrial length in (a) (n≥5, ***, P<0.001).

**c, d** DRP1-K38A could not further inhibit neural degeneration induced by polybrene in the presence of EGTA or nifedipine. The fraction of beads (**c**) or fragmented neurites (**d**) in neurons expressing DRP1-K38A or FLAG after treatment with polybrene for 12 h (n≥3, *, P < 0.05, ***, P < 0.001).

**SI Fig. 4: Mitochondrial dysfunction in neurites treated with polybrene.**

**a, b** mPTP opens during neuritic degeneration induced by polybrene: (**a**) Measurement of mPTP opening in neurons expressing mito-DsRed after polybrene treatment for 24 h. Cells were stained with calcein in the presence of CoCl_2._ Scale bar: 10 μm. (**b**) Quantification of calcein fluorescence. Fluorescence intensity of control was normalized to 1 (n≥10, ***, P<0.001).

**c, d** ΔΨ_m_ dissipation during neuritic degeneration induced by polybrene: (**c**) The far-red fluorescent dye Dilc(5) was used to monitor ΔΨ_m_ of neurons after polybrene treatment for 24 h. Scale bar: 10 μm. (**d**) Quantification of the Dilc(5) fluorescence intensity. Fluorescence intensity of control was normalized to 1 (n≥10, ***, P<0.001).

**e-g** Mitochondrial motility is suppressed after polybrene treatment. (**e**) Kymographs of neuritic mitochondria in neurons after polybrene treatment for 24 h. The relative velocity of neuritic mitochondria is shown in (**f**) (mitochondrial velocity of control neurites was normalized to 1), and the fraction of stationary or mobile mitochondria of total neuritic mitochondria is shown in (**g**) (n≥9, ***, P<0.001).

**SI Fig. 5: Polybrene does not affect mitochondrial (a) and ER (b) morphology in glia cells. Representative images.** Scale bars: 10 μm.
